# *Periplaneta americana* Oligosaccharides Exert Anti-Inflammatory Activity through Immunoregulation and Modulation of Gut Microbiota in Acute Colitis Mice Model

**DOI:** 10.3390/molecules26061718

**Published:** 2021-03-19

**Authors:** Kaimin Lu, Jing Zhou, Jie Deng, Yangjun Li, Chuanfang Wu, Jinku Bao

**Affiliations:** Key Laboratory of Bio-Resource and Eco-Environment of Ministry of Education, College of Life Sciences, Sichuan University, Chengdu 610065, Sichuan, China; lu_kaimin@126.com (K.L.); j17742878805@163.com (J.Z.); 2018141241104@stu.scu.edu.cn (J.D.); 18982404708@163.com (Y.L.)

**Keywords:** inflammatory bowel disorder, *Periplaneta americana*, oligosaccharide, gut microbiota, immune, oxidative stress

## Abstract

The incidence and prevalence of inflammatory bowel disorders (IBD) are increasing around the world due to bacterial infection, abnormal immune response, etc. The conventional medicines for IBD treatment possess serious side effects. *Periplaneta americana* (*P. americana*), a traditional Chinese medicine, has been used to treat arthritis, fever, aches, inflammation, and other diseases. This study aimed to evaluate the anti-inflammatory effects of oligosaccharides from *P. Americana* (OPA) and its possible mechanisms in vivo. OPA were purified and biochemical characterization was analyzed by HPGPC, HPLC, FT-IR, and GC–MS. Acute colitis mice model was established, the acute toxicity and anti-inflammatory activity were tested in vivo. The results showed OPA with molecular mass of 1.0 kDa were composed of 83% glucose, 6% galactose, 11% xylose, and the backbone was (1→4)-Glcp. OPA had potent antioxidant activities in vitro and significantly alleviated the clinical symptoms of colitis, relieved colon damage without toxic side effects in vivo. OPA exhibited anti-inflammatory activity by regulating Th1/Th2, reducing oxidative stress, preserving intestinal barrier integrity, and inhibiting TLR4/MAPK/NF-κB pathway. Moreover, OPA protected gut by increasing microbial diversity and beneficial bacteria, and reducing pathogenic bacteria in feces. OPA might be the candidate of complementary and alternative medicines of IBD with low-cost and high safety.

## 1. Introduction

Inflammatory bowel disorder (IBD) usually shows clinical symptoms of bloody diarrhea, rectal bleeding, bellyache and tenesmus, weight loss, abdominal pain, and cramping [1,2], which is associated with the significant reductions of patients’ quality of life and daily functioning [3]. Recently, the incidence and prevalence of IBD has been increasing around the world [4]. Consequently, IBD has been regarded as a public health issue and constantly impacted the medical system around the world.

Up to now, the precise pathogenesis of IBD remains unknown [5]. However, it is believed that both exogenous and endogenous factors are involved, such as genetic, infectious, immunologic, and colonic environment [6,7]. Currently, 5-aminosalicylates (5-ASA), biological, immunomodulators, and corticosteroids are the commonly used medicines, which have serious side effects such as anti-antibody reaction, allergy, infection and mutagenesis, fever, rash, fracture, or kidney problems [8]. Therefore, searching for novel therapeutic agents with higher effectiveness and less side effects is of great significance and urgency.

Nature products have good potentialities of being cost-effective benefit with high safety, such as artemisinin, taxol, and camptothecin [9]. Some plant polysaccharides exhibit efficacy in treating IBD, such as noni fruit [10] and *Arctium lappa* polysaccharide [11]. In addition, the medicinal insects, whose species are more than twice as many as medical plants, have also received extensive research interests [9]. *Periplaneta americana* (*P. americana*), a traditional Chinese medicine being present for over 300 million years, is widely distributed in tropical areas [9]. *P. americana* extract (PAE) has been used to treat arthritis, aches, pains, inflammation, chronic heart failure [12], cutaneous wounds [13], and other diseases for hundreds of years. Kangfuxin, the ethanol extract of *P. americana,* is approved by China Food and Drug Administration and has been used to treat various skin or mucosa injuries for over 40 years [14], which contains peptides, polyols, saccharide, amino acids, and other active substances [15]. However, the increased demand of Kangfuxin produces large amounts of *P. americana* residues after the ethanol extraction process. Currently, few studies pay attention to the utilization of *P. americana* residues. *P. americana* residues contained the high content of polysaccharides [14]. The low molecular weight and simple structure of oligosaccharides increase the original biological activity of polysaccharide [16,17], and have better bioavailability [18,19]. It is worth studying whether the oligosaccharides from *P. Americana* (OPA) had anti-inflammatory effects. In this study, OPA were separated and purified, its biochemical characterization, antioxidant activities in vitro, and anti-inflammatory effect in vivo were explored. Furthermore, its mechanism of anti-inflammatory was explored. These findings may pave a new way for developing effective drugs for IBD treatment and provide the basis for further analysis of OPA in the future.

## 2. Materials and Methods

### 2.1. Materials and Chemicals

The *P. americana* residues were collected from the Sichuan Gooddoctor-Panxi Pharmaceutical Company (Mianyang, Sichuan Province, China).

### 2.2. Separation and Purification of OPA

The powder of *P. americana* residues was obtained by grinding with a universal pulverizer (Tianjin Taisite Instrument Co., Tianjin China). The *P. americana* powder was extracted with PBS buffer (pH 6.5) in a ratio of 1:10 (*w*/*w*) at 560 w for 60 s in single-mode microwave-assisted synthesis equipment (Monowave 300, Anton Parr, Graz, Austria), and then extracted with 10,000 u papain papaya (Solarbio, Beijing, China) at 65 °C for 4 h. After enzyme inactivation at 90 °C for 2 min, centrifuged at 8000 g for 10 min. A 1/4 volume of 10% trichloroacetic acid was added to the supernatant to remove protein at 4 °C for overnight. After centrifuging at 8000 g for 10 min, the supernatant was separated by gel filtration chromatography with Sephacry S-100 high resolution column (1.5 × 95 cm, GE, American). OPA was dissolved in normal saline at 10 mg/mL. The injection volume was 2 mL, and the flow rate was controlled at 0.2 mL/min. The third peak was collected, concentrated and lyophilized for further research. The molecular mass of OPA was analyzed by comparison with the elution time based on the standard curve of a series of molecular mass standards (3.3, 6.5, 14.4, 20.1, and 31 kDa).

### 2.3. Biochemical Characterization of OPA

#### 2.3.1. Molecular Weight and Homogeneity Determination

The molecular weight of OPA was measured by HPGPC TSK-gel G-3000PWXL column (7.8 × 300 mm). OPA was dissolved in distilled water at 5 mg/mL. The injection volume was 20 μL, and elution was performed 0.2 M NaCl at a flow rate of 0.5 mL/min with a column temperature of 40 °C.

#### 2.3.2. FTIR Analysis

The OPA (1 mg) were ground with KBr, and determined on an infrared spectrometer (Perkin Elmer 1600 spectrometer) from 4000 to 400 cm^−1^. The functional groups of the polysaccharides were preliminarily analyzed by observing the absorption peaks.

#### 2.3.3. Monosaccharide Composition Analysis

Monosaccharide composition was determined with HPLC, following the methods of Wang et al. [11]. The 20 mg OPA samples were hydrolyzed into monosaccharide with 1 mL of absolute methanol contained 1 M hydrochloric acid, and the hydrolysates were dried by nitrogen blowing. Then, samples were hydrolyzed with 2 M trifluoroacetic acid (TFA) by incubation by incubation at 120 °C for 1 h, and removed to TFA completely at 60 °C. The hydrolysate solution was mixed with 0.5 mL 1-phenyl-3-methyl-5-pyrazolone (PMP) and 0.3 mL NaOH solution 70 °C for 30 min. Next, 200 μL hydrolysates were mixed with 100 μL of 0.3 M HCl solution and 100 μL distilled water. Chloroform was then added to extract superfluous PMP, and the samples were filtered through 0.22 μm membrane prior to further analysis. The monosaccharide composition was analyzed using Agilent Eclipse XDB-C18 column (4.6 × 250 mm). The injection volume was 10 μL, and elution was performed 81 (0.1 M, pH 7.0, PBS):19 (acetonitrile) (*v*/*v*) at a flow rate of 1 mL/min at 245 nm.

#### 2.3.4. Methylation Analysis of OPA

Methylation analysis was determined with GC–MS, following the methods of Ciucanu and Kerek [20]. OPA samples (5 mg) were dissolved in 1 mL DMSO under nitrogen atmosphere. The dissolved samples were mixed with 1 mL NaOH-DMSO solution, and stirred for 10 min. Then, 1 mL CH3I was added to the mixture in ice-cold water bath, and stirred for 30 min. The reaction was terminated with the addition of 2 mL distilled water, and the residue was extracted with CH_3_Cl. The extract was washed with distilled water and evaporated to dryness. The methylated polysaccharide was hydrolyzed with 1 mL 2 M TFA at 120 °C for 3 h. Ethyl alcohol was then added to extract TFA, and then mixed with 35 mg/mL NaBH_4_ for 12 h to reduce, then acetylated with pyridine and acetic anhydride. Acetylated samples were analyzed by GC–MS.

### 2.4. Determination of Antioxidant Activities

Ferric reducing power, hydroxyl and DPPH radical scavenging capacities were assessed according to the methods of Dhingra et al. [21], Zhao et al. [22], and Ciucanu and Kerek [20] respectively. The 1 mL samples (50–1000 μg/mL) were prepared and analyzed. Ascorbic acid (VC) was used as a positive control. The hydroxyl and DPPH radical scavenging activity was calculated at 510 and 517 nm, respectively, as follows:

Scavenging activity (%) = [A_0_ − (A_x_ − A_x0_)]/A_0_ × 100% (1)

where A_x_ was absorbance of the mixture with sample solution, A_0_ was absorbance of the mixture without sample; A_x0_ was absorbance of the mixture without chromogenic reagents.

### 2.5. Anti-Inflammation Activity

#### 2.5.1. Animals

Male 8-weeks-old KM mice and C57BL/6 mice were purchased from Dashuo Laboratory Animal Technology Co. in Chengdu of China. All animal experiments were performed according to the guidelines for Animal Care and Use Committee of China. Prior to the experiments, the mice were housed for at least 1 week at 25 °C under 12 h light/dark cycles with access to pellet food and water.

#### 2.5.2. Acute Toxicity Test

KM mice were randomly divided into two groups: the control group and OPA group (*n* = 6). The OPA group was fed orally at doses of 4000 mg/kg, the control group received the isometric saline solution for 7 days. All mice were weighed every day and kept under regular observation for any mortality or behavioral changes [23]. Then, they were sacrificed quickly by euthanasia and the serum was collected for evaluating cytokines. The liver, kidney, heart, spleen, thymus, and colon were weighed and the organ index was calculated. The oxidative stress capacities, including superoxide dismutase (SOD), malondialdehyde (MDA), catalase (CAT), and glutathione peroxidase (GSH-Px) in liver, were measured by ELISA kits (Mlbio, Shanghai, China).

#### 2.5.3. Establishment of Acute Colitis Mice Model

The C57BL/6 mice were randomly divided into control group, model group, low-dose OPA group, high-dose OPA group, and positive group (sulfasalazine) (*n* = 6). Mice of the normal group were given drinking water, and mice of other groups were fed 3% dextran sulfate sodium (DSS) water for 7 days [11]. Mice of the normal group and model group were given saline solution by intragastric administration for 14 days. Mice of low-dose OPA group, high-dose OPA group, and positive group were treated orally with 100 mg/kg OPA, 300 mg/kg OPA and 50 mg/kg sulfasalazine for 14 days, respectively. Disease activity index (DAI) and body weight were recorded every two days. DAI was determined as the sum of body weight, diarrhea, and bloody stool scores according to the scoring system in Table 1 [24]. Mice were sacrificed and serum was collected on day 15. Liver and colon were collected, and stored at 80 °C for further analysis. Mice feces were collected for intestinal microbiota analysis.

#### 2.5.4. Measurement of Cytokines

Colon was homogenated using RIPA buffer and protein inhibitor cocktail. The homogenates were kept on ice for 30 min and centrifuged at 3000 g for 15 min at 4 °C. Protein concentration was performed using the BCA Protein Assay Kit (NCM, Suzhou, China). The levels of IL-2, IL-4, IL-10, TNF-α, INF-γ in serum and colon were measured by ELISA kit (Mlbio, Shanghai, China).

#### 2.5.5. Histology Analysis

Colon tissue was fixed in 4% paraformaldehyde and embedded in paraffin. The embedded tissue was cut into 4 μm thin sections, and stained with hematoxylin and eosin (H&E). The histological changes were observed and the sections were scored according to Table 2 [25]. The histology scoring of colitis was based on the feature of inflammation, mucosal injury, crypt damage, and percent involvement.

#### 2.5.6. Immunohistochemistry Staining

For immunohistochemistry, colon sections were incubated with antibodies against zonula occludens-1 (ZO-1), Occludin, Claudins, or NLRP3 (Affinity Biosciences, Jiangsu, China). Then, colon sections were incubated with HRP-conjugated secondary antibody (Affinity Biosciences, Jiangsu, China). The signal was detected by DAB peroxidase substrate kit (Affinity Biosciences, Jiangsu, China). The results of immunohistochemical staining were visualized under Eclipse E100 Nikon light microscope (Nikon, Tokyo, Japan). The number of positive macrophages was counted by Image J.

#### 2.5.7. Measurement of Oxidative Stress

The liver and colon tissues were homogenized, and protein concentration was performed by the BCA Protein Assay Kit (NCM, Suzhou, China). The levels of SOD, MDA, CAT, and GSH-Px in liver and myeloperoxidase (MPO) in colon were measured by ELISA kit (Mlbio, Shanghai, China).

#### 2.5.8. Western Blot

The colon tissues were homogenized, and protein concentration was performed using the BCA Protein Assay Kit (NCM, Suzhou, China). Then, 25 µg proteins were separated with 12% SDS-PAGE gel and then transferred to PVDF membrane. The membranes were incubated with primary antibodies against toll-like receptor 4 (TLR4), P-p65, p65, P-p38, p38, p-ERK1/2, ERK1/2, P-JNK, JNK, or GAPDH (Affinity Biosciences, Jiangsu, China), followed by incubation with secondary antibodies. The signals were developed using an ECL Western blot detection kit (Affinity Biosciences, Jiangsu, China) and analyzed by Image J.

#### 2.5.9. Microbial Community Analysis

Mice feces were collected and stored at −80 °C. The DNA of total bacteria in mice feces was extracted with QIAamp^®^ Fast DNA Stool Mini Kit. The V4 region of the 16S rRNA gene was amplified using the barcoded primers. The PCR products were purified using the Qubit 2.0 (Thermo Fisher) and sequenced using the Illumina MiSeq platform. All the results were based on sequenced reads and operational taxonomic units (OTUs).

### 2.6. Statistical Analysis

Results were expressed as mean ± SEM. Differences between groups were determined by *t*-test using GraphPad Prism version 5. A value of *p* < 0.05 was considered to be statistically significant and *p* < 0.01 was considered to be extremely significant.

## 3. Results

### 3.1. Purification and Biochemical Characterization of OPA

**Figure 1:**
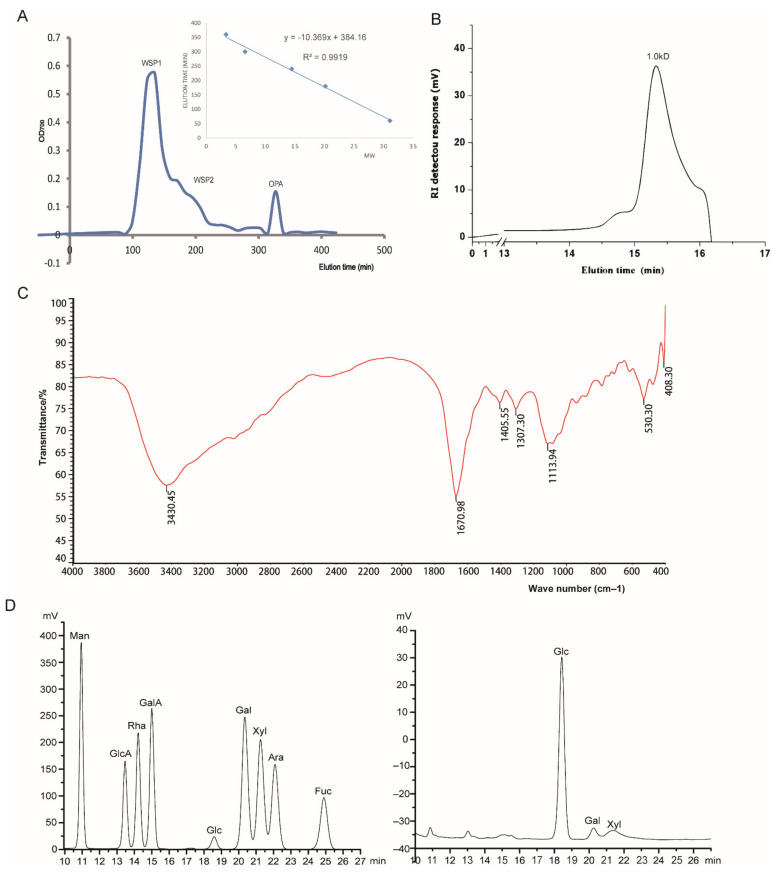
Purification and structural characterization of OPA. (**A**) Purification of OPA using gel filtration chromatography and an elution curve of a molecular mass standard; (**B**) HPGPC chromatography of OPA; (**C**) FT-IR spectrum of OPA; (**D**) the monosaccharide composition of OPA by HPLC; the left was the peak of monosaccharide standerds, the right was the peak of OPA.

The proteins were removed from the crude polysaccharides that were separated by gel filtration chromatography. Figure 1A shows three main peaks eluted by normal saline, designated WSP1, WSP2, and OPA, respectively. OPA peak (small molecular mass) was desalted and freeze-dried for further analysis.

The result of homogeneity showed that the molecular mass of peak in 15.329 min was 1.0 kDa (Figure 1B). As shown in Figure 1C, the FT-IR spectrum of OPA revealed that the strong and broad absorption around 3430 cm^−1^ was the characteristic peak of vibration of –OH due to stretching. The absorbance at 1652 cm^−1^ was attributed to the bond stretching vibrations of the carboxylate (C=O) bonds. The absorbance at 1113.94 cm^−1^ corresponded to the C-O stretching vibrations, which was the mark of saccharides. It was observed that the absorption peak of the α-type or β-type glycosides bonds of the polysaccharide at 760 or 870 cm^−1^ showed weak absorption.

The monosaccharide compositions of OPA were analyzed by HPLC. As shown in Figure 1D, OPA were composed of 83% glucose, 6% galactose, and 11% xylose. The glycosidic linkage was performed by methylation analysis. As summarized in Table 3, the methylation analysis of OPA consisted of 74% 1,4-Glcp, 2.9% 1,6-Glcp, 5.6% 1,4,6-Glcp, 7.8% 1-Glcp, and 9.7% 1-Xyl. These results suggested that the main backbone of OPA was 1,4-Glcp with 7% branching degree.

### 3.2. Antioxidant Activities In Vitro

**Figure 2:**
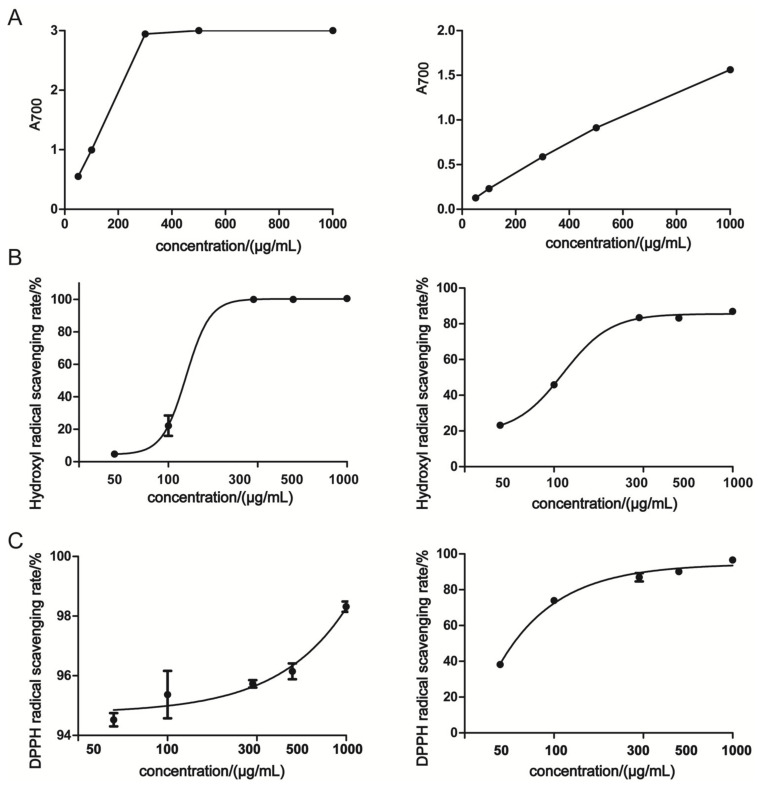
The antioxidant activities of OPA in vitro. (**A**) Ferric reducing power; (**B**) hydroxyl radical scavenging; (**C**) DPPH radical scavenging activity. The left was VC and the right was OPA. A_700_ was the absorbance value at 700 nm.

The antioxidant activities of OPA were determined by Ferric reducing power and free radical scavenging (hydroxyl and DPPH) assays. As shown in Figure 2A, the ferric reducing power of OPA was positively correlated with the concentration. The reducing power of OPA was lower than that of VC. The hydroxyl radical scavenging activity of OPA was dose-dependent, and its 50% free radical scavenging (IC_50_) was 112.3 µg/mL (Figure 2B, Table 4). The concentration responding to IC_50_ of VC was 125.6 µg/mL, which was higher than OPA. DPPH is widely used to estimate free radical scavenging activity of antioxidants in vitro. As seen in Figure 2C, the DPPH radical scavenging activity of OPA or VC was in a dose-dependent manner. The IC_50_ of DPPH radical scavenging activity of OPA was 61.56 µg/mL (Table 4). These findings suggested OPA exhibited potent antioxidant activities in vitro.

### 3.3. Acute Toxicity

**Figure 3:**
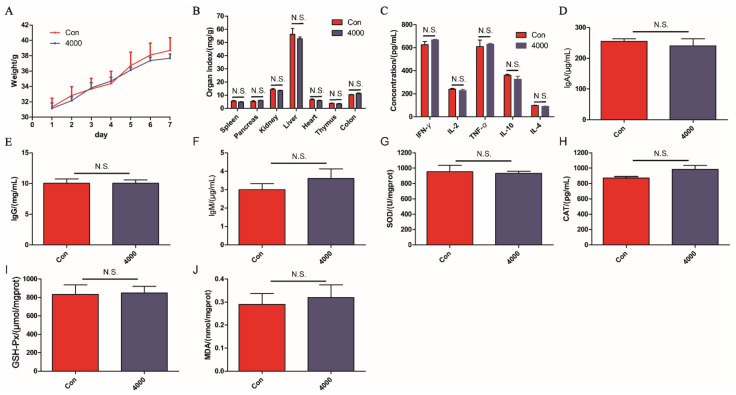
The acute toxicity of OPA in vivo. The large dose OPA affected weight (**A**) and organ index (**B**), cytokines (IFN-γ, IL-2, TNF-α, IL-10, IL-4) (**C**) and immunoglobulins (IgA, IgG, IgM) (**D**–**F**) in serum, oxidative stress indexes (SOD, CAT, GSH-Px, MDA) in liver (**G**–**J**). Con was control group, 4000 was 4000 mg/kg OPA group. Data were expressed as the mean ± SD (*n* = 6), the statistical analyses were done with *t*-test. N.S. was no significance.

During the supplement of OPA, the mice did not exhibit any mortality, abnormal behavior, and toxic symptoms. As shown in Figure 3A–J, the body weights, organ index (spleen, pancreas, kidney, liver, heart, thymus, and colon), the levels of cytokines (IFN-γ, IL-2, TNF-α, IL-10, IL-4) and immunoglobulin (IgA, IgG, IgM) in serum, and the levels of oxidative stress indexes (SOD, CAT, GSH-Px, and MDA) in liver were not significantly different between OPA treatment group (4000 mg/kg) and control group (*p* > 0.05). These results indicated that the OPA were safe and exhibited no toxic effect even at a high dose in mice.

### 3.4. Intestinal Protective Activity

#### 3.4.1. OPA Treatment Ameliorated DSS-Induced Colitis

**Figure 4:**
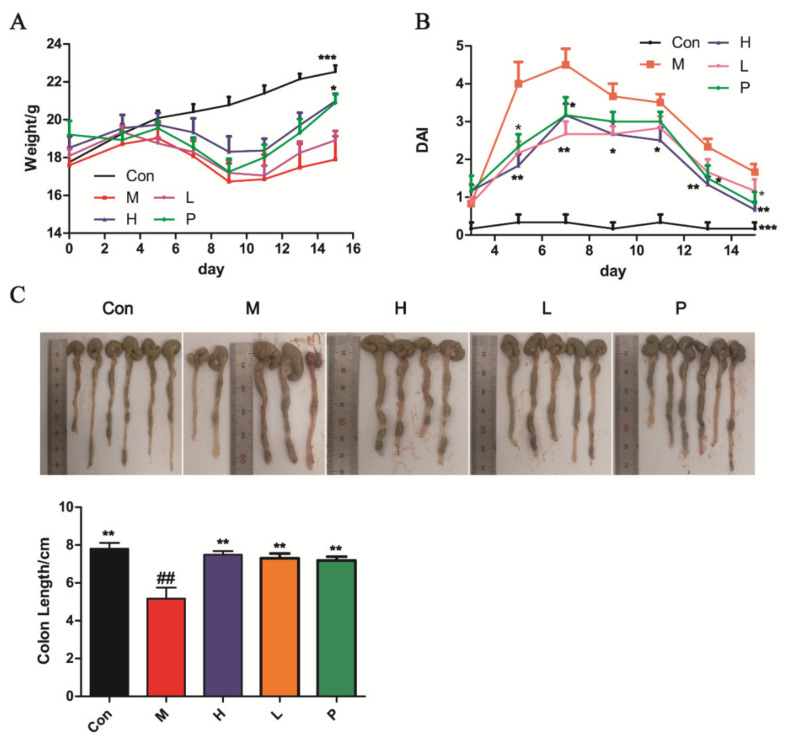
OPA alleviated the clinical symptoms of DSS-induced colitis. (**A**) The change of body weight during 14 days; (**B**) DAI; (**C**) colon length. Con was control group, M was model group, H was the high dose OPA group, L was low dose OPA group, P was positive group. Data were expressed as the mean ± SD (*n* = 6), the statistical analyses were done with *t*-test; *** *p* < 0.001, ** *p* < 0.01, * *p* < 0.05 compared with the model group; ^##^ *p* < 0.01, ^#^ *p* < 0.05 compared with the control group.

DSS treatment induced a marked decrease in body weight (*p* < 0.001, Figure 4A) and colon length (*p* < 0.01, Figure 4C). Meanwhile, the DAI score increased rapidly (Figure 4B). The administration of OPA or sulfasalazine reduced the body weight loss in colitis mice compared with model group mice (*p* < 0.05, Figure 4A). At the same time, the DAI score prominently decreased after treatment with OPA or sulfasalazine (*p* < 0.05, Figure 4B). On day 15, the colon length of treatment with OPA or sulfasalazine was not significantly different compared with the control group (*p* > 0.05, Figure 4C). These results indicated that OPA treatment alleviated the clinical symptoms of DSS-induced colitis.

#### 3.4.2. OPA Regulated Immune in DSS-Induced Colitis

**Figure 5:**
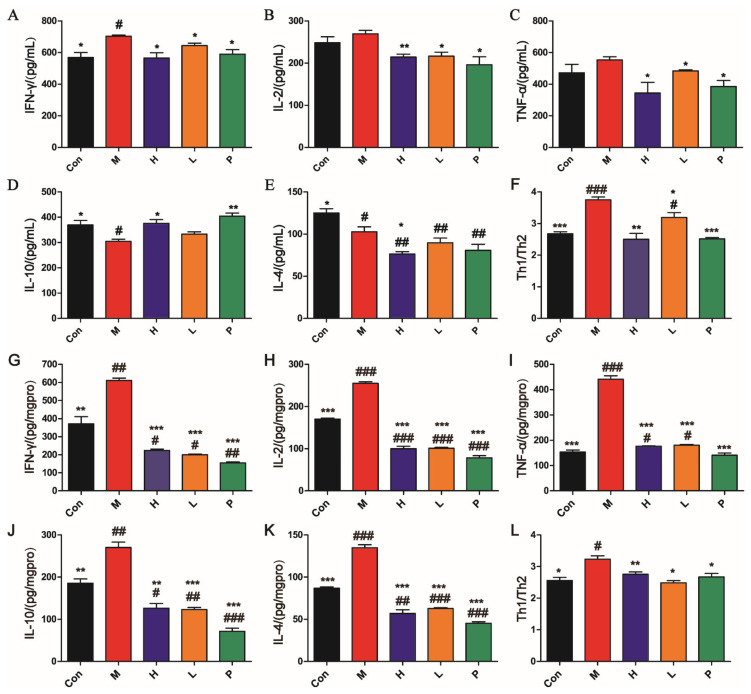
Effects of OPA on cytokines in serum (**A**–**F**) and colon tissues (**G**–**L**). Levels of IFN-γ (**A**), IL-2 (**B**), TNF-α (**C**), IL-10 (**D**), IL-4 (**E**), and Th1/Th2 (**F**) in serum and levels of IFN-γ (**G**), IL-2 (**H**), TNF-α (**I**), IL-10 (**J**), IL-4 (**K**), and Th1/Th2 (**L**) in colon tissues. Con was control group, M was model group, H was the high dose OPA group, L was low dose OPA group, P was positive group. Data were expressed as the mean ± SD (*n* = 6), the statistical analyses were done with *t*-test; *** *p* < 0.001, ** *p* < 0.01, * *p* < 0.05 compared with the model group; ^###^ *p* < 0.001, ^##^ *p* < 0.01, ^#^ *p* < 0.05 compared with the control group.

The contents of cytokines in serum are displayed in Figure 5A–E. Compared with control group, proinflammatory cytokines (IFN-γ) were markedly elevated (*p* < 0.05) and anti-inflammatory cytokines (IL-4 and IL-10) levels in serum were reduced in model group (*p* < 0.05). After administration of OPA or sulfasalazine, the levels of cytokines (IFN-γ, IL-2, TNF-α, and IL-4) were notably reduced compared with model group (*p* < 0.05). However, the level of IL-10 (anti-inflammatory cytokine) was observably elevated (*p* < 0.05). As depicted in Figure 5G–K, compared with control group, proinflammatory cytokines (IFN-γ, IL-2 and TNF-α) and anti-inflammatory cytokines (IL-4 and IL-10) levels in colon tissues were prominently elevated in the model group (*p* < 0.01). After administration of OPA or sulfasalazine, the levels of cytokines (IFN-γ, IL-2, TNF-α, IL-10, and IL-4) were significantly reduced compared with the model group (*p* < 0.001).

The ratio of Th1 (IFN-γ, IL-2 and TNF-α)/Th2 (IL-4 and IL-10) is the core pillar of immune balance of the body. This ratio is closely related to the malignant degree of acute colitis. As seen in Figure 5F,L, the Th1/Th2 ratio in the model group was observably higher than in the control group (*p* < 0.05). OPA or sulfasalazine regulated the aberrant Th1/Th2 ratio back to normal. These findings suggested that OPA suppressed DSS-induced inflammation by immunomodulatory effects.

#### 3.4.3. Histology Analysis

**Figure 6:**
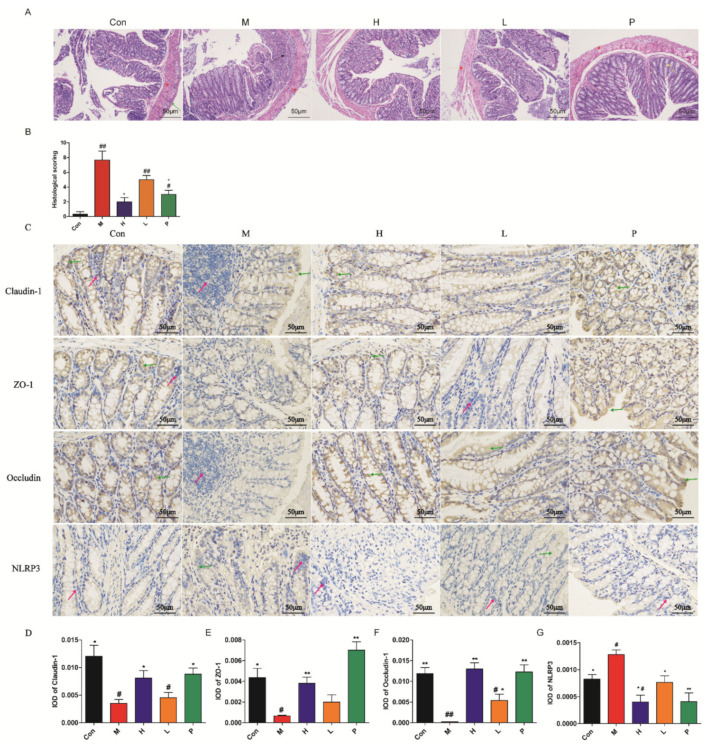
Colon histopathological and immunohistochemistry analysis. (**A**) H&E stain of colon tissues; red arrow was muscular layer, black arrow was inflammation cells, green arrow was serosal layer, blue arrow was mucous layer, yellow arrow was gland hyperplasia; (**B**) the histology scoring of colon histology; (**C**) effects of OPA on claudin-1, ZO-1, occludin, and NLRP3 of colon tissue in colitis mice; The integrated optical density (IOD) of claudin-1 (**D**), ZO-1 (**E**), occluding (**F**), and NLRP3 (**G**) positive cells. The red arrows represent nucleus; the green arrows represent positive cells. Con was control group, M was model group, H was the high dose OPA group, L was low dose OPA group, P was positive group. Data were expressed as the mean ± SD (*n* = 3), the statistical analyses were done with *t*-test; ** *p* < 0.01, * *p* < 0.05 compared with the model group; ^##^ *p* < 0.01, ^#^ *p* < 0.05 compared with the control group.

The pathology of colon, including inflammation, mucosal injury, crypt damage, and intactness of surface epithelium are displayed in Figure 6A,B. The histology analysis of colon tissues in control group showed a normal pathological morphology, no inflammatory response and damage. The histopathological analysis of colon tissues in the model group showed acute colitis pathological morphology, manifested as destroyed mucosal, the glands replaced by connective tissue, and numerous inflammatory cells. The histology analysis of colon tissues in OPA or positive group exhibited intact mucosal layer and crypt structure, and reduced numbers of inflammatory cells. As seen in Figure 6B, compared with control group, the histology scoring of colon tissues in the model group was markedly higher (*p* < 0.01), while the histology scoring of colon tissues in OPA group was not significantly different (*p* > 0.05). The colon tissues in OPA-treated mice showed a prominent decrease in pathological damage (*p* < 0.05).

#### 3.4.4. Immunohistochemistry Characterization

The immunohistochemistry characterizations of caludin-1, ZO-1, occludin, and NLRP3 are depicted in Figure 6C–G. Compared with control group, the levels of caludin-1, ZO-1, and occludin in the model group were observably reduced (*p* < 0.05, *p* < 0.05, *p* < 0.01, Figure 6C–F). Compared with the model group, the contents of tight junctions (TJs) protein in OPA or sulfasalazine treatment group exhibited notably elevated (*p* < 0.05). As seen in Figure 6G, compared with control group, the level of NLRP3 in colon tissues of the model group was markedly elevated (*p* < 0.05). The OPA or sulfasalazine could dramatically reduce the contents of NLRP3 in colon tissues (*p* < 0.05, *p* < 0.01). These results indicated OPA or sulfasalazine promoted intestinal barrier function and decreased inflammation.

#### 3.4.5. OPA Suppressed Oxidative Stress

**Figure 7:**
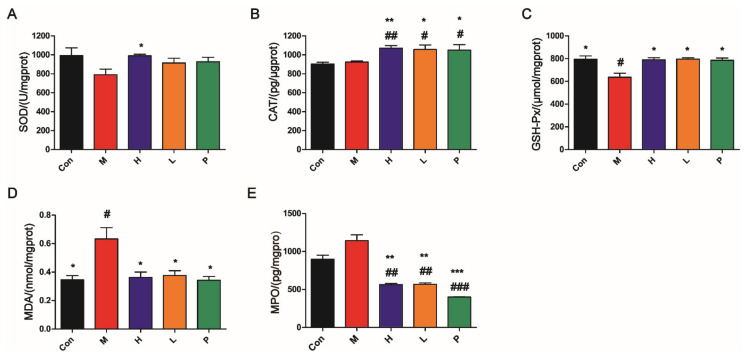
OPA suppressed oxidative stress in vivo. (**A**–**C**) Effects of OPA on SOD (**A**), CAT (**B**), and GSH-Px (**C**) (antioxidant enzyme) in liver; (**D**) effects of OPA on MDA (maker of oxidative stress) in liver; (**E**) effects of OPA on MPO in colon. Con was control group, M was model group, H was the high dose OPA group, L was low dose OPA group, P was positive group. Data were expressed as the mean ± SD, the statistical analyses were done with *t*-test; *** *p* < 0.001, ** *p* < 0.01, * *p* < 0.05 compared with the model group; ^###^ *p* < 0.001, ^##^ *p* < 0.01, ^#^ *p* < 0.05 compared with the control group.

As shown in Figure 7A–C, compared with the model group, the levels of antioxidant enzyme (SOD, GSH-Px, and CAT) in liver tissues of OPA or sulfasalazine treatment group were significantly increased (*p* < 0.05). On the contrary, the levels of MDA (maker of oxidative stress) in liver tissues and the levels of MPO (maker of inflammatory) in colon tissues of OPA or sulfasalazine treatment group were notably reduced (*p* < 0.05, *p* < 0.01, Figure 7D–E). These findings suggested that OPA or sulfasalazine had potential efficacy of improving antioxidant defense and reducing oxidative stress.

#### 3.4.6. OPA Inhibited TLR4/MAPK/NF-κB Signaling Pathway

**Figure 8:**
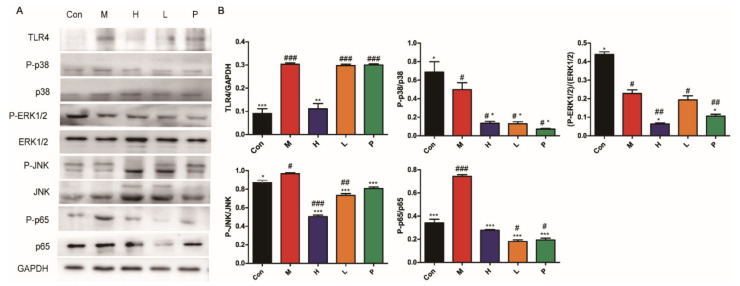
Inhibition of TLR4/MAPK/NF-κB signaling pathway. (**A**) Expression of TLR4, phosphorylated p38 (P-p38), p38, P-ERK1/2, ERK1/2, P-JNK, JNK, P-p65, p65 was shown. (**B**) The analysis of grey scale value. Con was control group, M was model group, H was the high dose OPA group, L was low dose OPA group, P was positive group. Data were expressed as the mean ± SD, the statistical analyses were done with *t*-test; *** *p* < 0.001, ** *p* < 0.01, * *p* < 0.05 compared with the model group; ^###^ *p* < 0.001, ^##^ *p* < 0.01, ^#^ *p* < 0.05 compared with the control group.

The expressions of important proteins in TLR4/MAPK/NF-κB signaling pathway are depicted in Figure 8. High dose of OPA inhibited the expression levels of TLR4 (*p* < 0.01), and OPA or sulfasalazine significantly inhibited the phosphorylation of MAPK (*p* < 0.001, JNK; *p* < 0.05, ERK1/2; *p* < 0.05, p38) and NF-κB (*p* < 0.001, p65). These results indicated the anti-inflammatory effects of OPA via suppressing TLR4/MAPK/NF-κB signaling pathway.

#### 3.4.7. Effects of OPA on Gut Microbiota

**Figure 9:**
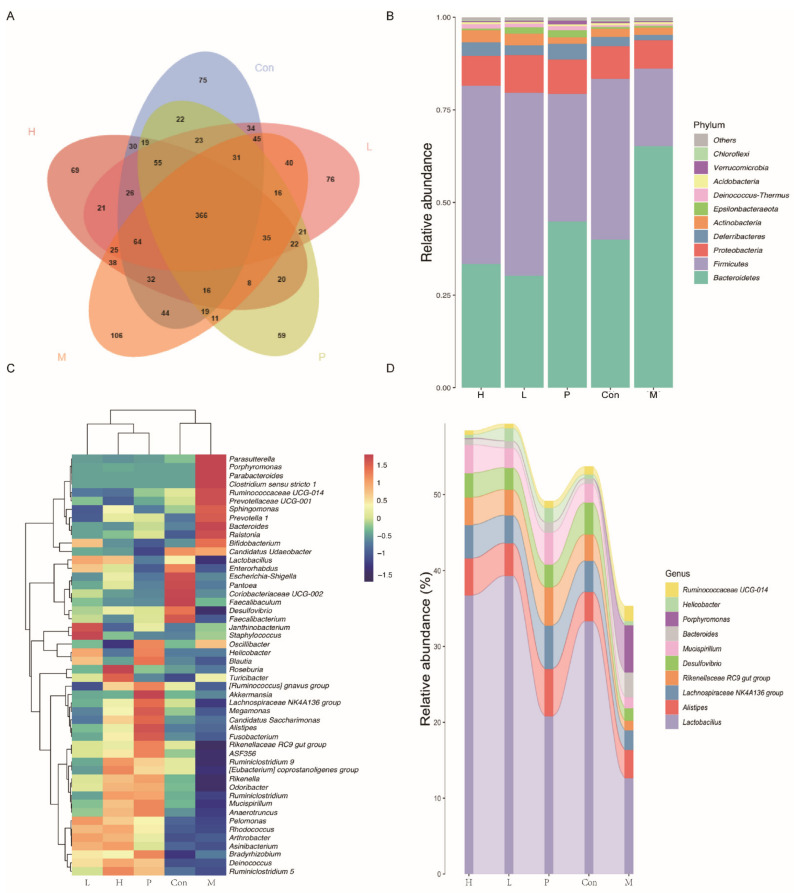
Effects of OPA on gut microbiota. (**A**) Venn chart; (**B**) the relative abundance of gut microbiota at phylum level; (**C**) the relative abundance of gut microbiota at genus level; (**D**) the analysis of sankeyplot of high abundance genus and random forest.

The relative abundance at phylum level and genus level, sankeyplot of high abundance genus are shown in Figure 9. Venn diagram (Figure 9A) showed the logical connection between different groups. Compared with model group, the ratio of *Fimicutes/Bacteroidetes* in OPA or sulfasalazine treatment group was observably increased, which indicated that OPA or sulfasalazine could promote efficient absorption of calories from food (*p* < 0.05, Figure 9B). In model group, the levels of *Parasutterella, Porphyromonas, Parabaceterdrides, Clostridium sensu stricto 1, Ruminococcaceae UGG-014, Precotellaceae UGG-001, Sphingomonas, Prevotella, Bacteroides*, and *Ralstonia* were increased (Figure 9C). Among these, the *Clostridium sensu stricto 1* and *Prevotella* were pathogenic bacterium, which produced toxin and then triggered inflammation. In OPA treatment group (Figure 9C), the levels of *Esulfovibrio, Faecalibacterium, Lachnospiraceae NK4A136 group, Megamonas, Rikenellaceae RC9 gut group, ASF356, Ruminiclostirdium 9, [Eubacterium] coprostanoligenes group, Rikenella, Odoribacter, Ruminiclostridium, Mncispillum*, and *Anaerotruncns* were increased. The metabolites of F*aecalibacterium, [Eubacterium] coprostanoligenes group, Odoribacter*, and *Anaerotruncns* had anti-inflammatory effects. As seen in Figure 9D, compared with model group, the levels of beneficial bacterium were increased, such as *Lactobacllius*, *Desulforibrio*, and *Rikenellaceae RC9 gut group*. These results showed that OPA protected the gut by increasing the amount of beneficial bacterium and decreasing the amount of pathogenic bacterium.

**Figure 10:**
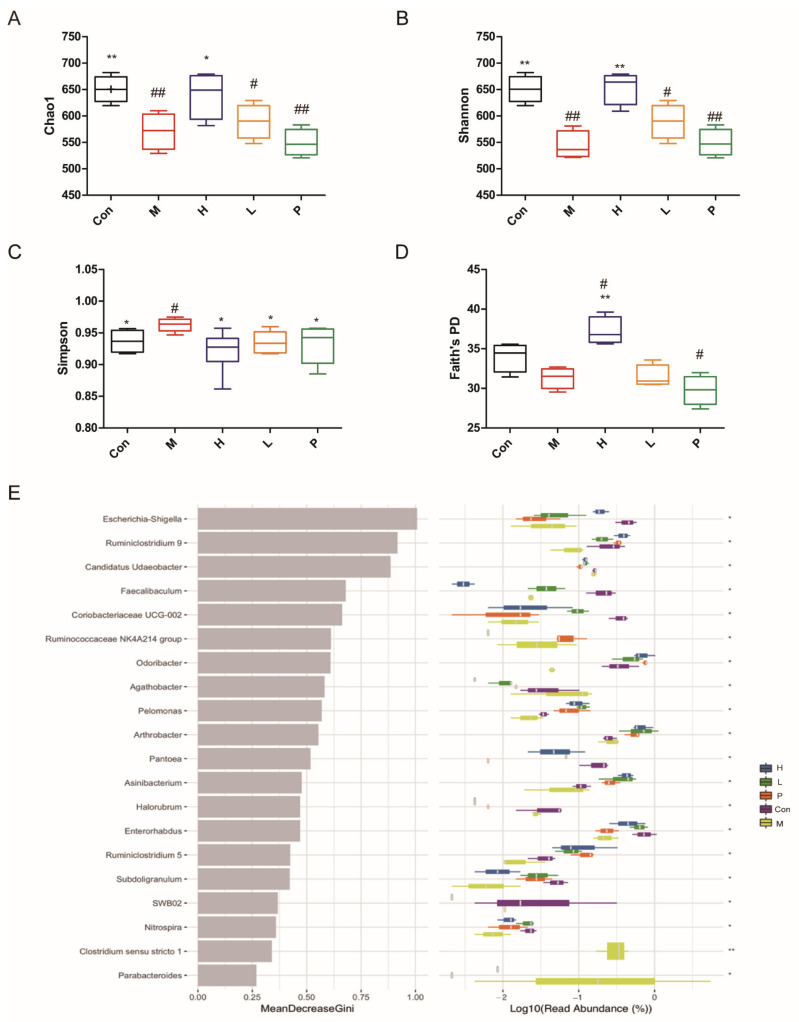
Effects of OPA on the alpha diversity of gut microbiota. The indexes of Chao1 (**A**), Shannon (**B**), Simpson (**C**), Faith’s PD (**D**) of gut microbiota; the random forest analysis of gut microbiota (**E**). Con was control group, M was model group, H was the high dose OPA group, L was low dose OPA group, P was positive group. Data were expressed as the mean ± SD, the statistical analyses were done with *t*-test; ** *p* < 0.01, * *p* < 0.05 compared with the model group; ^##^ *p* < 0.01, ^#^ *p* < 0.05 compared with the control group.

The alpha diversity and random forest analysis are shown in Figure 10. The alpha diversity represented by Chao1, Shannon, Simpson, and Faith’s PD is displayed in Figure 10A–D. Compared with the control group, the indexes of Chao1 and Shannon in the model group were prominently reduced (*p* < 0.01), and the indexes of Simpson in the model group were increased (*p* < 0.05). Compared with the model group, the indexes of Chao1, Shannon and Faith’s PD in high dose of the OPA treatment group were observably increased (*p* < 0.05, *p* < 0.01, *p* < 0.01), and the indexes of Simpson in the OPA treatment group were reduced (*p* < 0.05), which indicated OPA increased the diversity of gut microbiota. The random forest analysis is shown in Figure 10E, the microbiota that had high values of mean decrease Gini was important in the classification among groups. These findings indicated that OPA increased the alpha diversity of gut bacterium.

## 4. Discussion

DSS is the most widely used chemical drug for the establishment of the colitis model [25,26]. DSS treatment disrupts intestinal epithelial barrier, inhibits crypt cell proliferation and promotes apoptosis, causing diarrhea, rectal bleeding, body weight loss, hematochezia, colon length shortening, crypt abscess, and inflammation, which resembles human ulcerative colitis [5,27]. *P. americana* possesses excellent therapeutic effects with a long clinical application history [28,29], such as antitumor, enhanced immunity, antibacterial, anti-inflammatory and analgesic effects, tissue repair [30] and promoting liver regeneration [31]. PA-40, an extract of *P. americana*, has been widely used in the treatment of ulcerative colitis in China, and has achieved good results [30]. Periplanetasin-2 [32] and periplanetasin-5 peptide [33], derived from *P. americana*, exert significant anti-inflammatory activities in Clostridium difficile toxin A induced pseudomembranous colitis mice and lipopolysaccharides (LPS)-induced Raw264.7 macrophage cells, respectively. In the present study, we demonstrated that OPA prevented DSS-induced colitis with lower-cost and higher safety.

The intestinal barrier is a crucial defense system, which can resist the invading pathogens, endogenous microorganisms, and toxins. Chronic inflammation in IBD is caused by epithelial barrier function impairment [34,35], which facilitates the access of antigens from the intestinal lumen and generates dysregulation of intestinal mucosal immunity. TJs and intestinal epithelial cells (IECs) are the main components of the intestinal barrier [26]. The dysfunctions of IECs and TJs induced colonic inflammation. Accordingly, maintaining the integrity of physical barrier is crucial for treatment IBD [36]. Occluding, claudin-1, and ZO-1 are major TJ proteins. OPA increased the expression of TJ proteins. These findings suggested that OPA maintained physical barrier by restoring the impaired intestinal TJs and keeping epithelial integrity.

The imbalance of T lymphocyte subsets (Th1/Th2) leads to a disordered intestinal immune system and colonic tissue damage in IBD [37]. The proinflammatory cytokines are increasingly expressed in the inflamed gut in IBD patients and DSS-treated mice [38]. The secreted proinflammatory cytokines (IFN-γ, IL-2 and TNF-α) from immune cells result in loss of intestinal epithelial barrier integrity and magnify inflammatory response in colitis [39,40]. IL-10 inhibits the expression of other inflammatory cytokines produced by activated monocytes or macrophages [41,42]. The results showed OPA decreased the contents of proinflammatory cytokines (IFN-γ, IL-2 and TNF-α), conversely, increased the content of anti-inflammatory cytokines (IL-10). These findings suggested that OPA modulated the immune response via balancing Th1/Th2 and then improved the symptoms of IBD in vivo.

Oxidative stress could disrupt the intestinal barrier and then exaggerate the mucosal inflammatory response [43]. The damaged antioxidant balance, immoderate oxygen free radicals and reactive oxygen species conduce to excessive oxidative stress in IBD [44]. Lipid peroxidation could damage the intestinal epithelial cells and activate inflammatory mediators [45]. MDA is an indication of oxidative damage and a marker for free radicals-induced lipid peroxidation [46]. SOD, GSH-Px, and CAT are main antioxidant enzymes in the body. It has been reported that there is a significant increase in activity of MPO in colon tissues of DSS-treated mice [47]. MPO is abundantly secreted in neutrophils, and used as a marker for the influx of neutrophils as well as inflammation [48,49]. NLRP3 is a primary mediator of DSS-induced colitis. OPA observably decreased the activity of MPO and the content of NLRP3 in colon tissues. Moreover, OPA had antioxidant activity in vitro and OPA reduced the level of MDA, and elevated the level of antioxidant enzymes (SOD, GSH-Px, and CAT). These results indicated that OPA attenuated inflammation via antioxidant activity.

Immune system, oxidative stress and signal pathway could form an interactive network. Immune dysfunction and over-oxidation could lead to the destruction of the physical barrier and inflammation in IBD [26,43]. Reactive oxygen species (ROS) act as a messenger of intracellular signaling transduction and activate NF-κB, which causes and aggravates the inflammatory response in IBD [50,51]. TLR4/MAPK/NF-κB pathway is the main mediator of inflammatory responses, which plays crucial roles in immune and inflammatory response [52]. TLR4 is an important pattern recognition receptor, which is activated by saccharides and subsequently activates MAPK and NF-κB pathways [53]. Once MAPK and NF-κB have been activated, they can be translocated to the nucleus for regulating the activity of AP-1 proteins, which regulate many proinflammatory cytokines and enzymes, such as IL-1β, IL-6, iNOS, COX-2, and TNF-α [54,55,56]. Additionally, NF-κB is activated in the IBD patients and contributes to pathogenesis of IBD [57]. PAE can reduce inflammatory by inhibiting MAPK/NF-κB cascade [12]. Chitosan oligosaccharides (COS) suppressed NF-κB activation, attenuated proinflammatory cytokine production in IBD [41]. Thus, downregulating the activity of NF-κB and MAPK is the candidate way for treatment of IBD. The results suggested that OPA attenuates inflammation in vivo by downregulating the TLR4/MAPK/NF-κB pathway, which suggested that OPA could be natural antioxidants and immunomodulators.

Gut microbiota plays a key role in immune and inflammatory functions [58]. The decline of beneficial bacteria and/or an increase in pathogenic bacteria in gut promotes intestinal microbiota imbalance in IBD, which is characterized by a reduction of bacterial diversity and an increase in the ratio of *Bacteroidetes*/*Firmicutes* [59,60]. The *Bacteroidetes*/*Firmicutes* ratio was usually used for assessing the composition of gut microflora [60,61,62]. The shift ratio suggested that OPA protected against inflammatory in IBD. Recent reports have suggested that prebiotic oligosaccharides (such as galacto-oligosaccharides or fructo-oligosaccharides, psyllium, COS) have a beneficial effect that keeps a healthy colon by regulating the growth and differentiation of colonocytes in the treatment of IBD [63,64,65]. The prebiotic oligosaccharides may play a role in increasing the production of short-chain fatty acids (SCFAs), and reducing mucosal damage, inflammatory cells infiltration [61,66]. Studies have shown that SCFAs can renew anti-inflammatory and immunoregulatory activity and restore the intestinal mucosa after injury [43]. Increased SCFA levels could stabilize pH in colon and contribute to the maintenance of colonic homeostasis [66]. In our study, OPA increased the beneficial bacteria which had anti-inflammatory effect by producing SCFAs, such as *Lactobacllius, Desulforibrio, Rikenellaceae RC9 gut group, Desulfovibri, Faecalibacterium, [Eubacterium] coprostanoligenes group, Odoribacter,* and *Anaerotruncns*, and reduced the level of pathogenic bacteria, including *Porphyromonas, Clostridium sensu stricto 1, Prevotellaceae UGG-001, Bacteroides,* and *Ralstonia*. *Bacteroides* can produce cellular endotoxin LPS which activate the TLR4/MAPK/NF-κB pathway. Thus, OPA could downregulate the TLR4/MAPK/NF-κB pathway by reducing the level of *Bacteroides*. The results showed that OPA increased microbial diversity and beneficial bacteria, and reduced pathogenic bacteria in feces. All these results indicated that OPA attenuated inflammation by improving the structure of gut microbiota and downregulated the TLR4/MAPK/NF-κB pathway.

The prebiotic oligosaccharides can block the progress of inflammatory responses [67]. Konjac oligosaccharide (1–4 g/kg/day) is an anti-inflammatory and could be useful as a prebiotic to design functional foods for ulcerative colitis [44]. Oral administration of feruloylated oligosaccharides (400 mg/kg/day) effectively alleviates mice colitis disease induced by DSS [38]. COS administration (10–20 mg/kg/day) significantly alleviates the intestinal inflammation in both DSS-induced colitis and rectal acetic acid-induced colitis model [68]. OPA (300 mg/kg/day) exhibited anti-inflammatory activity in DSS-induced colitis model mice. The oligosaccharides increase the original biological activity due to their structural characteristics and low molecular weight. OPA showed antioxidant activity and anti-inflammatory effects might be relative to the low molecular weight and the reactive groups, such as −OH, C=O, which had antioxidant capacity.

## 5. Conclusions

In conclusion, OPA with a molecular mass of 1.0 kDa was composed of glucose, galactose, and xylose, and the backbone was (1→4)-Glcp. We verified OPA had anti-inflammatory activity by modulating immune, reducing oxidative stress, inhibiting TLR4/MAPK/NF-κB pathway, preserving intestinal barrier integrity, and regulating gut microbiota with high safety in vivo. These findings may pave a new way for developing effective drugs that can be applied in IBD treatment in the future.

## Figures and Tables

**Table 1:** Disease activity index (DAI) scoring system.

Score	Weight Loss (%)	Diarrheal Stool Score	Bloody Stool Score
0	<1	Normal	Negative
1	1–5	--	--
2	5–10	Loose	Positive
3	10–15	--	--
4	>15	Diarrhea	Gross bleeding

**Table 2:** Histological scoring system.

Score	Inflammation	Mucosal Injury	Crypt Damage	Percent Involvement (%)
0	None	None	None	0
1	Slight	Mucous layer	1/3	1–25
2	Moderate	Submucosal	2/3	26–50
3	Serious	Muscular layer and serosa	1	51–75
4	--	--	1 + epithelial loss	76–100

**Table 3:** Methylation analysis of OPA.

O-Me-Alditol Acetate	Linkages	Percent (%)
2,3,6-Me3-Glcp	1,4-Glcp	74
2,3,4-Me3-Glcp	1,6-Glcp	2.9
2,3-Me2-Glcp	1,4,6-Glcp	5.6
2,3,4,6-Me4-Glcp	1-Glcp	7.8
2,3,4,6-Me4-Xylp	1-Xyl	9.7

**Table 4:** IC_50_ values of hydroxyl radical scavenging and DPPH radical scavenging.

IC_50_	VC	OPA
IC_50_ of hydroxyl radical scavenging (µg/mL)	125.6 ± 1.445	112.3 ± 1.292
IC_50_ of DPPH radical scavenging (µg/mL)	<<50	61.56 ± 0.893

## Data Availability

Data presented in this study are available on request from the authors.

## Sample Availability

Samples of OPA are not available from the authors.

## Abbreviations

The following abbreviations are used in this manuscript:

5-ASA	5-aminosalicylates
CAT	Catalase
COS	Chitosan oligosaccharides
DAI	Disease activity index
DSS	Dextran sulfate sodium
GSH-Px	Glutathione peroxidase
H&E	Hematoxylin and eosin
IBD	Inflammatory bowel disorder
IECs	Intestinal epithelial cells
IOD	Integrated optical density
LPS	Cellular endotoxin lipopolysaccharides
MDA	Malondialdehyde
MPO	Myeloperoxidase
OPA	Oligosaccharides from *P. Americana*
OTUs	Operational taxonomic units
*P. Americana*	*Periplaneta Americana*
PAE	*P. americana* extract
PMP	Phenyl-3-methyl-5-pyrazolone
ROS	Reactive oxygen species
SCFAs	Short-chain fatty acids
SOD	Superoxide dismutase
TFA	Trifluoroacetic acid
TJs	Tight junctions
TLR4	Toll-like receptor 4
VC	Ascorbic acid
ZO-1	Zonula occludens-1
